# Risk factors of Non-fatal Unintentional Home Injuries among Children under 5 Years Old; a Population-Based Study

**Published:** 2017-01-08

**Authors:** Sedigheh Nouhjah, Sharareh R. Niakan Kalhori, Azadeh Saki

**Affiliations:** 1 Social Determinants of Health Research Center, school of Health, Ahvaz Jundishapur University of Medical Sciences, Ahvaz, Iran.; 2 Health Research Institute, Diabetes Research Center, Ahvaz Jundishapur University of Medical Sciences, Ahvaz, Iran.; 3 Department of Health Information Management, School of Allied Medical Sciences, Tehran University of Medical Sciences, Tehran, Iran.; 4 Department of Biostatistics, School of Health, Ahvaz Jundishapur University of Medical Sciences, Ahvaz, Iran.

**Keywords:** home injuries, child, preschool, risk factors, injury prediction, risk reduction

## Abstract

**Introduction:**

In addition to the annual mortality rate, unintentional home injury may result in temporary or permanent disability and requires medical attention and continuous care in millions of children. This study aimed to explore features and risk factors of these injuries.

**Methods:**

In this cross-sectional study, demographic variables and epidemiologic pattern of home injuries among children under 5 years of age were collected via a population-based survey in seven main cities of Khuzestan province, southwest Iran, during September 2011 to December 2012. Developing a risk stratification model, independent risk factors of unintentional home injury were determined and put to multivariate logistic regression analysis.

**Result:**

2693 children with the mean age of 27.36 ± 15.55 months (1 to 60) were evaluated (50.9% boy). 827 (30.7%) cases had a history of at least one home injury occurrence since birth to study time. The most common injury mechanisms were burning with 291 (38.4%) cases, falling with 214 (28.3%) and poisoning with 66 (8.7%) cases, respectively. The independent risk factors of unintentional home injury were age ≥ 24 month (p<0.001), residency in Ahvaz city (p<0.001), mother’s illiteracy (p<0.014), ethnicity (p<0.001), private housing (p=0.01), birth weight (p<0.001), and being the first child (p=0. 01). Sensitivity, specificity, and area under the ROC curve of the model designed by multivariate analysis were 53.5%, 84.8%, and 0.75 (95% CI: 0.73- 0.77; P < 0.001, figure 1), respectively.

**Conclusion:**

According to the findings of this study, 30.7% of the studied children were injured at least once since birth. Burning, falling, poisoning, swallowing objects, choking, and biting were the main home injury mechanisms. Age ≥ 24 months, being the first child, living in a private house, being a resident of Ahvaz city, and having an illiterate mother were found to be risk factors of home injury.

## Introduction

Alteration in epidemiological pattern of childhood mortality, progress in control of infectious diseases, and increased risk of injuries have created a new view of child mortality and morbidity in the world (1). Injury is a major cause of death from childhood to 10-19 years old (1, 2). In addition to the annual mortality rate, injury may result in temporary or permanent disability and requires medical attention and continuous care in millions of children (3). About 95% of child injuries and more than 80% of related deaths occur in low and middle-income countries (1, 4). About 90% of child deaths resulting from injuries or violence, occur due to unintentional injuries (1).

Unintentional injuries may influence health, education, and family economy of the affected children (3). Most unintentional childhood injuries take place in the home, where children spend a long period of their time and are supposed to be well supervised; however, they are exposed to various injury hazards (5-7). The most common injury mechanisms have been reported as falling, burning, swallowing, poisoning, choking, biting and drowning in a variant order (3, 8). In the current care system, the same process is used to prevent every type of injury mechanism among children under the age of 5 years, which is mainly brief education for their mothers. Screening children who are at a higher risk of home injury based on their epidemiologic features is a fantastic idea for preventing home injuries, efficiently. Children can be classified based on their potential risk of home injury and their mother can then be educated on factors that might categorize the child as prone to a specific injury mechanism. Currently, there is a lack of patterns related to child injury and available researches are mainly hospital-related works. Since only a limited number of child home injuries lead to hospitalization, this type of investigation may ignore small and limited emergencies. Middle or small scale injures that need temporary therapeutic interventions may not be registered anywhere and recently this limitation has received more attention than before. Thus, this population-based study aimed to explore features and risk factors of non-fatal unintentional home injuries among children under 5 years old in Khuzestan province of Iran.

**Table1:** Frequency of home injuries based on demographic features of less than 5 year's children and their mother

**List of Variables**	**Total n** (%)	**History of home injury n (%)**		**P Value**
		**Yes**	**No**	
**Gender**				
Male	1369 (50.9)	409(29.9)	960(70.1)	0.33
Female	1322 (49.1)	418(31.6)	904(68.4)	
**Age Group (month)**				
0-12	584 (21.7)	119(20.4)	465(79.6)	<0.001
13-24	830 (30.8)	240(28.9)	590(71.1)	
25-36	504 (18.7)	176(34.9)	328(65.1)	
37-48	483 (17.9)	177(36.6)	306(63.4)	
49-60	291 (10.8)	115(39.5)	176(60.5)	
**City**				
Ahvaz	900 (33.4)	327 (36.3)	573(63.7)	<0.001
Andimeshk	299 (11.1)	77(25.8)	222(74.2)	
Behbahan	300 (11.1)	55(18.3)	245(81.7)	
Khoram-Shahr	299 (11.1)	157(52.5)	142(47.5)	
Ramshir	300 (11.1)	72(24.0)	228(76.0)	
Shoush	300 (11.1)	51(17.0)	249(83.0)	
Baghmalek	295 (11.0)	88(29.8)	207(70.2)	
**Mother’s Job**				
Employed	159 (5.9)	45(28.3)	114(71.7)	0.50
Housekeeper	2521 (94.1)	777(30.8)	1744(69.2)	
**Mother’s literacy**				
Illiterate	202 (7.6)	71 (35.1)	131(64.9)	0.014
Primary School	611 (22.9)	208(34.0)	403(66.0)	
Middle School	701 (26.2)	200(28.5)	501(71.5)	
High School	886 (33.2)	275(31.0)	611(69)	
Academic	271 (10.1)	65(24.0)	206(76.0)	
**Mother’s Ethnicity**				
Arab	1285 (49.0)	472(36.7)	813(63.3)	<0.001
Fars	523 (20.0)	130(24.9)	393(75.1)	
Lore	707 (27.0)	177(25.0)	530(75.0)	
Other	107 (4.0)	36(33.6)	71(66.4)	
**Type of house**				
Apartment	502 (26.0)	171(34.1)	331(65.9)	0.01
Private	1431 (74.0)	581(40.6)	850(59.4)	
**Birth order**				
1	1343 (50.0)	438(32.6)	905(67.4)	0.01
2	859 (32.0)	248(28.9)	611(71.1)	
3	322 (12.0)	86(26.7)	236(73.3)	
≥ 4	163 (6.0)	54(33.1)	109(66.9)	

**Table2:** Common mechanisms of home injuries based on demographic features of under 5 year’s children and their mothers

**Variable**	**Burning**	**Falling**	**Poisoning**	**Swallowing**	**Choking**	**Biting**	**Others**
**Gender**							
Male	151(39.7)	102(26.8)	30(7.9)	27(7.1)	15(3.9)	19(5.0)	36(9.5)
Female	140(37.1)	112(29.7)	36(9.5)	20(5.3)	14(3.7)	13(3.4)	42(11.1)
**Age group (month)**							
0-12	36(31.6)	29(25.4)	6(5.3)	22(19.3)	5(4.4)	6(5.3)	10(8.8)
13-24	82(38.1)	59(27.4)	19(8.8)	13(6.8)	14(6.5)	8(3.7)	20(9.3)
25-36	70(43.2)	49(22.9)	18(27.3)	3(6.0)	2(6.9)	5(15.6)	15(19.2)
37-48	63(38.9)	44(20.6)	15(22.7)	6(3.7)	5(17.2)	8(25.0)	21(26,9)
49-60	40(38.5)	33(15.4)	8(12.1)	3(2.9)	6(12.8%)	3(10.3%)	12(15.4)
**City of residence**							
Ahvaz	100(37.3)	37(13.8 )	30(11.2)	31(11.6)	11(14.1)	19(7.1)	40(14.9)
Andimeshk	17(22.1)	31(40.3)	9(11.7)	4(5.2)	10(13.0)	2(2.6)	4(5.2)
Behbahan	26(53.1)	11(22.4)	3(6.1)	0(0)	2(4.1)	0(0)	7(14.3)
Khoram-Shahr	75(48.1)	58(37.2)	4(2.6)	3(1.9)	4(2.6)	0(0)	12(7.7)
Ramshir	39(54.9)	16(22.5)	2(2.8)	6(8.5)	1(1.4)	2(2.8)	5(7.0)
Shoush	5(9.8)	24(47.1)	12(23.5)	0(0)	0(0)	7(13.7)	3(5.9)
Baghmalek	29(34.1)	37(43.5)	6(7.1)	3(3.5)	1(1.2)	2(2.4)	7(8.2)
**Mother’s Job**							
Employed	272(38.4)19	193(27.3)	65(9.2)	45(6.4)	26(3.7)	32(4.5)	75(10.6)
Housekeeper	(42.2)	19(42.2)	1(2.2)	2(4.4)	3(6.7)	0(0.0)	3(6.7)
**Mother’s literacy**							
Illiterate	24(34.8)	19(27.5)	7(10.1)	4(5.8)	3(4.3)	4(5.8)	8(11.6)
Primary School	88(45.4)	50(25.8)	13(6.7)	12(6.2)	4(2.1)	6(3.1)	21(10.8)
Middle School	62 (34.1)	60(33.0)	21(11.5)	6(3.3)	12(6.6)	9(28.1)	12(6.6)
High School	95 (38.8)	62(25.3)	21(8.6)	19(7.8)	7(2.9)	11(4.5)	30(12.2)
Academic	19(31.1)	21(34.4)	3(4.9)	6(9.8)	3(4.9)	2(3.3)	7(11.5)
**Mother’s ethnicity**							
Arab	180(41.5)	105(24.2)	37(8.5)	29(6.7)	14(3.2)	16(3.7)	53(12.2)
Fars	41(36.0)	33(28.9)	9(7.9)	10(8.8)	4(3.5)	6(5.3)	11(9.6)
Lore	50(30.7)	60(36.8)	18(11.0 )	8(4.9)	9(5.5)	8(4.9)	10(6.1)
Other	17(47.2)	12(33.3 )	2(5.6)	0(0)	2(5.6)	1(2.8)	2(5.6)
**Pregnancy type**							
Wanted	254(38.5)	187(28.4)	52(7.9)	40(6.1)	26(3.9)	27(4.1)	73(11.1)
Unwanted	34(37.0)	25(27.2)	13(14.1)	7(7.6)	3(3.3)	5(5.4)	5(5.4)

**Table 3:** Results of multivariate logistic regression analysis

**Risk Factors**	**Odd ratio (95%CI)**	**P value**
**Residency in Ahvaz city**	0.133 (0.07- 0.22)	< 0.001
**Age ≥ 24 month**	1.01 (1.00 – 1.02)	< 0.001
**Mother’s illiteracy**	2.09 (1.20- 3.40)	0.005
**Private housing**	3.19 (2.20 – 460)	< 0.001
**First child**	2.0 (1.28 – 3.12)	< 0.001

CI: Confidence interval.

**Figure 1:**
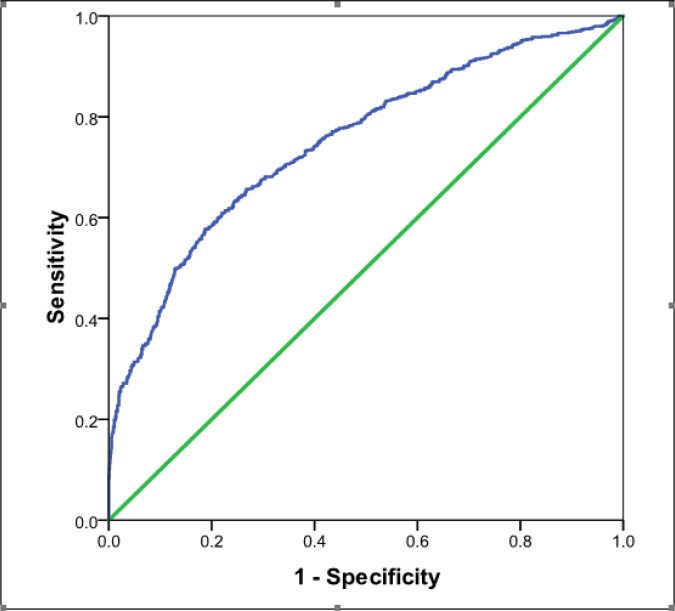
Area under ROC curve for probability of having unintentional home injury in children under 5 years.

## Methods

***Study design and setting***

In this cross-sectional study, data were collected via a population-based survey of home injuries for children under 5 years of age in seven main cities of Khuzestan province, southwest Iran, during September 2011 to December 2012. Selected cities included Ahvaz, Andimeshk, Khoramshahr, Shoush, Baghmalek, Behbahan, and Ramshir, which were the most populated places in terms of children under care. Ahvaz is the capital of Khuzestan province and has a number of different ethnic groups; the others are also main cities with different socioeconomic, cultural and ethnic groups. In each city 4-8 health centers were randomly selected based on covered population. The WHO guideline for external injury was used for coding and classifying the data. Before data collection, the study`s ethical approval was obtained from Ahvaz Jundishapur Medical University Ethical Research Committee under ethics number eth-684. In addition, the aim of study was explained to study cases and researchers adhered to data confidentiality.

**Participants**

Children under 5 years old seeking routine care were included using a multistage cluster sampling method. Their mothers were asked about their offspring`s experience of non-fatal unintentional home injuries since birth. Cases related to intentional home injuries such as interpersonal violence, domestic abuse, child abuse, self-inflicted harm, or crime, as well as injuries leading to death or occurring outside the home were excluded. 

**Data gathering**

Parent-related data (their age, job, education level, ethnicity), mother's pregnancy type (wanted, unwanted), family status (living with both parents or one of them, house type, number of children), Child’s features (age, birth weight, gender, birth order, age when injured), whether a child suffered an injury during the last year and since birth, injury details (mechanism, frequency, season, time and place, activity at the time of occurrence, hospitalization or physician visit, duration of hospitalization, physical/mental disability, injured organ, person responsible for event), were recorded in a 63-item questionnaire. It was designed using “WHO guideline for external injury” and confirmed through a pilot study conducted in Ahvaz (9). The content validity of the research tool was verified by two experts including pediatrician and a statistician. Its reliability was confirmed using Cronbach's alpha coefficient (0.81). Data were gathered by a group of trained public health students.

**Statistical analysis**

Data were analyzed using SPSS version 16. Qualitative variables were reported as frequency and percentage, and quantitative ones as mean ± standard deviation. In the first step, independent risk factors of unintentional home injury among children under 5 years old were determined using univariate analysis. Trying to develop a risk stratification model, independent variables of univariate analyses were put to multivariate logistic regression analysis. We used backwards elimination method for identifying risk factors of home injuries in 7 steps. Screening performance characteristics of the model were reported (sensitivity, specificity, and area under receiver operating characteristics (ROC) curve). P value < 0.05 was considered as level of significance.

## Results

2693 children with the mean age of 27.36 ± 15.55 months (1 to 60) were evaluated (50.9% boy). Table 1 summarizes baseline characteristics of studied population. 827 (30.7%) cases had a history of at least one home injury occurrence since birth to study time. The extremities were the injured part of body in more than 50% of cases and 68.8% (1852) of injuries occurred during playing. Common mechanisms of home injuries based on demographic features of children and mothers are summarized in table 2. The most common injury mechanisms were burning with 291 (38.4%) cases, falling with 214 (28.3%), poisoning with 66 (8.7%), swallowing with 47 (6.2%), biting with 32 (4.2%), and chocking with 29 (3.8%), respectively. 341 (69.5%) of injured children needed medical visits and 143 cases (39.4%) were hospitalized.

Based on univariate analysis, the independent risk factors of unintentional home injury among children under 5 years old were age ≥ 24 month (p<0.001), residency in Ahvaz city (p<0.001), mother’s illiteracy (p<0.014), ethnicity (p<0.001), private housing (p=0.01), birth weight (p<0.001), and being the first child (p=0. 01). Table 3 shows the results of multivariate logistic regression analysis. The model’s sensitivity, specificity, and area under the ROC curve were 53.5%, 84.8%, and 0.75 (95% CI: 0.73- 0.77; P < 0.001, figure 1), respectively. 

## Discussion

According to the findings of this study, 30.7% of the studied children were injured at least once since birth. Age ≥ 24 months, being the first child, living in a private house, being a resident of Ahvaz city, and having an illiterate mother were found to be risk factors of home injury among under 5 year old children. Burning, falling, poisoning, swallowing objects, choking, and biting were the main home injury mechanisms and the prevalence of home injuries did not vary by gender.

Children under five years old are at higher risk of home injuries as they spend more time at home compared to older children, and are unable to manage potential hazards due to their physical development stage. Other studies have also shown high prevalence of non-fatal injuries among children under 5 (10). In line with our study, Qiu et al. and Arif et al. also reported that children over 24 months old had a higher risk of home injury (11, 12). 

Regarding mechanism of injury, our results are in line with other studies that have reported burning, falling and poisoning as the most common mechanisms among preschool children (9, 11, 13). 

No gender differences were detected regarding injury risk, which is in line with Arif study (12). However, according to the National Child Development study, there is about two folds raise in risk for boys (14). In contrast, Chan et al. showed that female gender is associated with higher risk of home injuries (15). 

Children living in private houses were more at risk for injury, which might be due to the lifestyle in Khuzestan province particularly in cities such as Ramshir, Baghmalek and Shoush, Khoramshahr. A study reported that need for home repair correlated with injury risk (16). Since apartments are more recently built, this might be the reason that children living in private houses are at higher risk of injury. 

In this study, mother’s illiteracy and being the first child were risk factors of home injury. Both might be due to the fact that illiterate mothers and first time mothers have less information regarding handling and taking care of a child. In line with the results of this study, a study in Egypt also showed that birth order is a risk factors of home injury. In contrast, Addor et al. reported that birth order did not affect injury risk and Halawa et al. reported that 2^nd^ and 3^rd^ born children are at higher risk of injury (17, 18). In addition, regarding mother’s illiteracy they reported that children of highly educated mothers are at higher risk of injury (17). In line with the results of this study, Kamal reported that children of less educated parents were at higher risk for injury (19).

Higher frequency of non-fatal home injuries was detected among children of some specific ethnicity with low level of socio-economic living standard, low quality of housing, and low level of mother education level. Pregnancy type also strongly correlated with injuries and affected mother’s attitude to protecting and taking care of the children; reported also in other investigations(11, 20). In fact, there family socio-economical and maternal parenting and supervision level also have been addressed as determinants of non-fatal children injury occurrence (20, 21); challenging living conditions, lack of safe place to play as well as child care giver absence put children at the risk of injury (22).

Being a resident of Ahvaz city was also a risk factor for unintentional injuries. This is not consistent with the results of a meta-analysis that reported in Canada and the US rural children are more at risk for injuries (23). 

From injury prevention perspective, this study implies that the risk of non-fatal injury in children under 5 years old is a complex and multi-dimensional and successful prevention strategies setting require to target multiple components. Efforts to reduce the risk of home injury require a risk estimation tool to predict non-fatal home injuries. High risk families for child home injury occurrence should be identified by heath care providers through screening tools to help parents to classify their risky offspring. Health care providers or parents or both should develop different strategic teaching systems to effectively enhance children’s understanding of the safety issue in order to reduce children’s risk of hazard (24). We suggest further investigations to uncover more potential mechanisms and home causality predictors, which may enable classifying risky children more accurately.

## Limitations

This study had some limitations. Since the mother was asked about data regarding former injuries she might have either forgotten or lied about the child’s history to avoid being labeled as irresponsible. In addition, more attention to data collection to have balanced subgroups and evaluating more variables, such as attendance of another person at the time of injury occurrence, could improve level of model sensitivity. In addition, further research on home injury risk estimation in grades and scores are needed; this propose might define and estimate risk score more precisely rather than only a risky class. Application and comparison different intelligent modeling methods such as machine learning approaches as has been applied in other areas can be used both for more accurate models development and risk estimation purpose(25). These models have the potentiality of being replaced by current system that consider all children in same level of risk and provide the unique intervention for all children. 

## Conclusion:

According to the findings of this study, 30.7% of the studied children were injured at least once since birth. Burning, falling, poisoning, swallowing objects, choking, and biting were the main home injury mechanisms. Age ≥ 24 months, being the first child, living in a private house, being a resident of Ahvaz city, and having an illiterate mother were found to be risk factors of home injury. 
